# The Revival of the Notes Field: Leveraging the Unstructured Content in Electronic Health Records

**DOI:** 10.3389/fmed.2019.00066

**Published:** 2019-04-17

**Authors:** Michela Assale, Linda Greta Dui, Andrea Cina, Andrea Seveso, Federico Cabitza

**Affiliations:** ^1^K-tree SRL, Pont-Saint-Martin, Italy; ^2^University of Milano-Bicocca, Milan, Italy; ^3^Politecnico di Milano, Milan, Italy; ^4^Link-Up Datareg, Cinisello Balsamo, Italy; ^5^IRCCS Istituto Ortopedico Galeazzi, Milan, Italy

**Keywords:** natural language processing (NLP), literature review, machine learning, clinical intelligence, text mining, information extraction, data quality, sentiment analysis

## Abstract

**Problem:** Clinical practice requires the production of a time- and resource-consuming great amount of notes. They contain relevant information, but their secondary use is almost impossible, due to their unstructured nature. Researchers are trying to address this problems, with traditional and promising novel techniques. Application in real hospital settings seems not to be possible yet, though, both because of relatively small and dirty dataset, and for the lack of language-specific pre-trained models.

**Aim:** Our aim is to demonstrate the potential of the above techniques, but also raise awareness of the still open challenges that the scientific communities of IT and medical practitioners must jointly address to realize the full potential of unstructured content that is daily produced and digitized in hospital settings, both to improve its data quality and leverage the insights from data-driven predictive models.

**Methods:** To this extent, we present a narrative literature review of the most recent and relevant contributions to leverage the application of Natural Language Processing techniques to the free-text content electronic patient records. In particular, we focused on four selected application domains, namely: data quality, information extraction, sentiment analysis and predictive models, and automated patient cohort selection. Then, we will present a few empirical studies that we undertook at a major teaching hospital specializing in musculoskeletal diseases.

**Results:** We provide the reader with some simple and affordable pipelines, which demonstrate the feasibility of reaching literature performance levels with a single institution non-English dataset. In such a way, we bridged literature and real world needs, performing a step further toward the revival of notes fields.

## 1. Introduction

In recent years there has been a growth of the availability of medical data thanks to the increasingly wider adoption of Electronic Health Record (EHR) (1) in hospital settings. These massive quantities of data (known as “Big Data”) hold the promise of supporting a wide range of medical and healthcare functions, including clinical decision support, disease surveillance, and population health management (2).

As widely known, Big Data is not only a matter of volume: this term denotes the management of data sets that present a challenge in any dimensions related to the extraction of value out this process, like velocity (or volatility), veracity (or quality), and variety. This last dimension is the main relevant in characterizing free text, and other unstructured information produced in hospitals, such as biomedical signals and imaging.

An oft-mentioned (but never proved) conjecture affirms that 80 percent of data contained in EHRs are unstructured (3), that is it is recorded in “form of narrative text notes, either typed or dictated by physicians” (4). Unstructured content is then just the text of anamnestic notes, physical examination sheet, medical and nurse diaries, surgical forms, specialist test reports, and discharge letters written during the patient's stay, and any comment extending the standard patient-reported outcome (PRO) measures collected during the follow-up phase. Since this kind of heterogeneous textual content is created to support the primary purpose of care (5), it is less suitable to secondary uses [that is research- and administrative-oriented purposes (6)] than coded data filled in standard “structured” forms: it is therefore less prone to be queried than data stored in relational databases and it is characterized by an intrinsic multiplicity of expressions by which doctors and patient can report the same medical condition (7).

However, it is also well known that unstructured data contain relevant, and richly detailed and nuanced information about the illness trajectory and care processes undertaken by and upon the patients (8), and this makes the challenge to automatically extract accurate information from narrative notes worthy to be pursued (9). The fact that narrative content is used mainly to allow for practitioners' recall, and as a means for doctor-doctor communication over different work shifts (10), makes it substantially not affected by opportunistic manipulations or interpretation errors that affect some structured content (e.g., upcoding and misclassification) (11).

The main promise of Natural Language Processing (NLP) techniques in medicine is to achieve a good accuracy with respect to the manual review of (electronic) patient records and extraction of medical concepts and patient values (12), while requiring much lower amount of resources (time and money) with respect to this manual, time-consuming and often error-prone (13) task. NLP is nowadays an integral and established area of computer science in which machine learning (ML) and computational linguistics are broadly used (14). Although cutting edge NLP technologies, which employ the rules of linguistics, deep learning architectures or a combination of both these approaches, have been generally proved to be effective and sufficiently reliable with respect to user-generated content available on the Web (15), their application to medical content and hence biomedical research is relatively more recent and still susceptible of some improvements (4, 16). Indeed, compared to some traditional applications of NLP and Text Mining techniques, the analysis of the medical records shows further and specific difficulties, e.g., due to speciality- and setting-specific medical terminologies, the frequent use of abbreviations and jargon, as well as the difficulty of putting concepts into mutual relation.

Some comprehensive literature reviews have been recently published on the application of NLP to EHR unstructured content for various purposes and in different medical specialties [e.g., (17–20)]. For this reason, our study will not be aimed at reaching a similar comprehensiveness in the short span of time since the publication of the contributions mentioned above. Rather, we will focus on some specific applications of NLP techniques that we believe reach a good compromise between feasibility [since they do not require powerful computational means or difficult adaptation to different settings (9)] and short-term return, in that they have a potential to both improve data quality (11) and the accuracy and reliability of computerized decision support (21–23). This is the reason why we will not cover the application of NLP to realize either text- or voice-based conversational agents (24) and scribe-like transcription systems [e.g., (25)], or we will just hint at applications to build complex phenotipic data by which to train highly-accurate predictive systems (21, 26–28): these are both application domains that will certainly attract great interest in the next years but their real-life adoption is still in its infancy and limited to research groups that can get access to large and high-quality corpora of medical texts and patient interactions, like Google DeepMind^1^ and Amazon^2^.

Rather, we chose to focus on a few and well-circumscribed NLP applications to outline their main features and discuss their feasibility and cost-effectiveness in the real-world application to the surgery electronic registries adopted at IRCCS Istituto Ortopedico Galeazzi (IOG). IOG is a large teaching hospital of Northern Italy specializing in clinical research on locomotor disorders and associated pathologies, where almost 5,000 surgeries are performed yearly, mostly arthroplasty (hip and knee prosthetic surgery) and spine-related procedures, and are electronically documented on the DataReg system. This latter is an electronic pathology registry that stores together the structured data from the Admission-Discharge-Transfer system of the hospital and the followup PRO data with the unstructured content of the surgery diary and discharge letters.

In what follows we will present: an introduction to the field of NLP (section 2); then, we provide an overview of the main applications of NLP in clinical context related to some of the most relevant contributions in the recent literature (related works in section 3); then, we outline the main results of a small empirical study we implemented at IOG (empirical work in section 3), using part of the methodology explained in the related works; finally, we report the discussion (section 4) of these results and the conclusion (section 5) of this work.

## 2. A Brief Introduction to NLP

Natural Language Processing (NLP) is a subfield of computer science that tries to learn, understand and produce human language content (15). Natural Language (i.e., human language) means the language that we use in everyday life both written and spoken. It is so called to distinguish it from formal language, such as computer language. Actually, natural language is more complex because it can contain ambiguities and misunderstandings. For this reason, it is more difficult to process compared to the computer language. Research on NLP started in the 1950's when a group of researchers tried to implement an automatic translation from Russian to English (29). Concerning this first approach to NLP we can talk only of Machine Translation. Before the 1980's NLP approaches were based mainly on linguistics rules. It was only from the 1980's that, thanks to the increase in computational power, NLP problems started to be addressed by Machine Learning algorithms, and nowadays we have many approaches that can be combined in order to obtain reliable and robust results in NLP (29). Therefore, NLP is a part of Artificial Intelligence (AI) with an overlap with Linguistics. In particular, both Machine Learning (ML) and Deep Learning (DL) can be used to solve NLP challenges. In general, typical applications of NLP are Machine Translation, Automatic Summarization, Sentiment Analysis, and Text Classification^3^.

Natural languages are extremely complex systems. Similarly to human body, a human language has several sub-systems such as phonology, morphology, and semantics working seamlessly with each other (30):

1. Phonology means sound patterns;
2. Morphology includes characters and words;
3. Syntax comprises sentences, grammar and phrases;
4. Semantics involves words meaning, implication and logic.

The main steps of a typical NLP process flow are described below. First, it is necessary to collect a corpus of unstructured data; this step includes data mining that means the implementation of a process to identify patterns in large datasets and establish relationships to solve problems through data analysis (14). Then, a pre-processing step is needed, to ensure data accuracy, completeness and consistency. In this step, sentences are usually split into words (“Tokenization”), single words are converted in their base form (“Lemmatization,” for example verbs in past participle form are converted in their present form) and finally words are identified as nouns, adjectives, verbs etc (“Parts-of-Speech-tagging”)^3^. As third step, the pre-processed words need to be analyzed to assign them a meaning. This step is called feature engineering, which includes the conversion of text into a vector or array of numbers (‘Word Embedding’). Finally, different NLP algorithms can be used. NLP is usually associated with Machine Learning or Deep Learning algorithms. For example, classification algorithms can be applied for the detection of consumer sentiment. The more traditional approach to NLP is hand-crafted rules, formulated and compiled by linguists or knowledge engineers (14).

## 3. NLP Applications in Clinical Context

In order to explore the application of NLP in medicine we made a search mainly on Google Scholar using combined keywords such as NLP, EHR, text mining, medicine, clinical note, data quality, automated coding, named entity recognition, sentiment analysis, predictive models. Since NLP applied in medicine is a recent topic we concentrated in articles published in the last 5 years even if some older articles about the theme NLP are used. We focused on the papers published in the most important journals that deal with informatics applied to clinical context such as Journal of Medical Internet Research, JAMIA, Computer Methods and Programs in Biomedicine, Journal of biomedical informatics, International journal of medical informatics.

As above said, we explore some specific domains of NLP applications: data quality, information extraction, sentiment analysis and predictive models, and automated patient cohort selection.

Data quality is important for data reliability and validity, structuring text means to properly convert unstructured data into structured data to make them suitable for processing, sentiment analysis can be useful to extract information from data to build predictive models for diagnosis and prognosis and finally automated cohort selection is the automated detection of patients with specific features for epidemiologic studies or clinical trials.

An important aspect of the use of NLP in medicine related to the Data Quality domain is the automated summarization of EHRs and Electronic Medical Records (EMRs). Pivovarov and Elhadad (31) made a review that analyzes this topic and in particular focused on methods for detecting and removing redundancy, describing temporality, determining salience, accounting for missing data, and taking advantage of encoded clinical knowledge.

Concerning the second domain, a review study conducted by Yadav et al. (32) highlighted the importance of mining EHRs to improve patient health management since EHRs contain detailed information related to disease prognosis for large patient populations. For this purpose, a research group (33) tried to convert clinical texts into Unified Medical Language System (UMLS) code applying existing NLP system (MedLEE) with good results in terms of recall and precision. Moreover, NLP for entity extraction is also used (not widely used) in veterinary medicine for example in inferring diagnostic codes from free text notes (34).

A review conducted by Meystre et al. (35) established that much of the available clinical data are in narrative form as a result of transcription of dictations, direct entry by providers, or use of speech recognition applications. Therefore, sentiment analysis in medical context is important since physicians usually give a subjective interpretation in their diagnosis whereas NLP could offer a high-level text understanding by providing a more objective information (36). In general, after sentiment extraction from free-texts, it could be possible to build predictive models to help physicians in prognosis and diagnosis.

The aim of automated cohort selection is the extraction of data from EHRs to find inclusion/exclusion criteria to identify a cohort of patients to fit a specific clinical trial or to analyze specific features of patients (37).

### 3.1. Data Quality Assessment and Improvements

#### 3.1.1. Related Works

**Figure 1:**
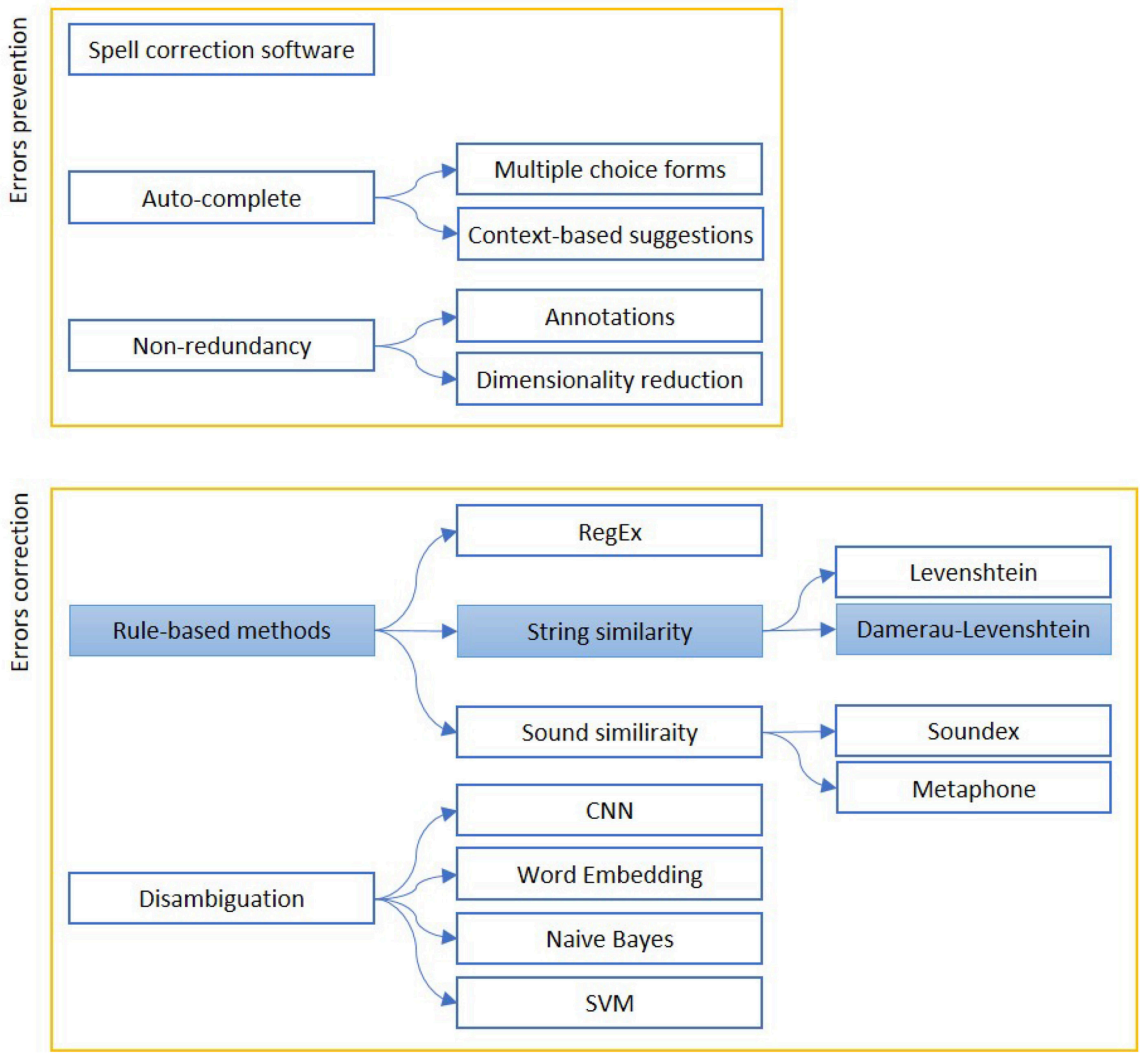
The figure shows the most relevant measures to assess (*Errors prevention*) and improve (*Errors correction*) the quality of a free written text. Errors prevention can be performed before errors are made (Auto-complete techniques), contextually (Spell-correction software) or with a simplification, to avoid redundant information insertion. Error correction can be based on string similarity, when it is a typing error, or sound similarity, when it is a spelling error. Disambiguation is used to find similar expression on a context-basis. The colored boxes highlight the experimental approach proposed in this article.

We report in Figure 1 a diagram that presents the most relevant methods related to the topic of “quality assessment and improvement” as a summary of the methods present in the literature and which we have reported in more detail below.

Data quality is the NLP application that aims to improve data reliability detecting transcription errors, words/sentences inconsistency, ambiguities in natural language and also additional noise from a variety of sources such as misspellings and abbreviations (38). Due to these problems the use of medical Big Data must proceed with caution because it is clear that NLP must deal with data quality problems and try to solve them (39).

As a matter of fact, a challenging aspect of NLP in medicine is the disambiguation of abbreviations. Joopudi et al. (40) trained a Convolutional Neural Network (CNN) to disambiguate abbreviation senses. For example, mg could have two senses: myasthenia gravis and milligrams. They used three datasets of which the first two were created from 1,001 longitudinal patient records they received from Cleveland Clinic, Ohio (USA), which contained a total of 117,526 clinical notes. The first one was automatically generated, the second one was manually annotated from the set-aside notes and the third dataset was a publicly available resource created by the team at University of Minnesota^4^ and contains 37,500 instances of 75 abbreviations with about 500 instances for each abbreviation. They finally assessed that the CNN model had the best performances (with an accuracy of 0.95) compared to more traditional approaches like Support Vector Machines (SVM) and Naïve Bayes (NB) in disambiguating words.

Moreover, a review made by Sun et al. (41) asserts that data in EHR and EMR are diverse, incomplete and redundant. For this reason, they identified several preprocessing steps to follow to make clinical data reliable and improve their accuracy. These steps include data cleansing, data integration, data reduction, data transformation and privacy protection. The first stage aims to make data cleaner in terms of noise, inconsistency and incompleteness. The second focuses on improving the speed and accuracy of data mining, dealing with heterogeneous data and its redundancy. Data reduction is used to improve efficiency reducing the size of the dataset (keeping the same information): these methods include dimension reduction, quantity reduction, and data compression. The fourth step refers to the conversion of dataset into a unified form suitable for data mining, including smoothing noise, data aggregation and data normalization. The last step, which is quite critical in medical care system, includes methods such as data encryption, privacy anonymity processing, and access control. For each of these steps the authors presented several examples of applications.

Concerning data reliability, a study by Knake et al. (42) wanted to solve this problem providing detailed guidelines that address specific issues in order to minimize EHR extraction errors. For example, a frequent issue is that the same parameter is recorded redundantly in different sections of the record, often by different caregivers; the derived guideline states that, when performing abstraction, only one value is picked. Another problem detected by the authors is related to the non-readily computability of EHR's data. The associated guideline suggests to perform manual abstraction. They focused mainly on structured data but they tried to settle also free-text data although NLP applications must typically be tailored to specific problems. They performed data extraction comparing manually and automated methods. The results showed that the discrepancy range observed with electronic extraction was comparable to the one obtained by manual abstraction.

Another group of researchers (43) stated that to obtain good results datasets should be properly annotated so they described a platform, built by DefineCrowd^5^, to create high-quality datasets in NLP and speech technologies domains. Such platform is based on a workflow that takes text or audio as the first input and the output of this first step becomes the input of the following step and so forth. The idea is to configure a ML service to pre-annotate the data, transforming the next step (done by humans) into a validation and correction of the ML service output. Finally, the different workflows enable to obtain a score which assesses the quality of the dataset.

In 2017, two Italian researchers, Marcheggiania and Sebastiani (44), devoted their studies to investigate the training data-quality effects on the learning process for the clinical domain. In particular, they focused on information extraction systems. They chose 2 annotators, one for training data labeling (annotator_B) and the other one for testing data labeling (annotator_A). They wanted to investigate how the annotators disagreement affected the accuracy of the classificator. The results showed that no statistically significant decrease was observed when the annotator_B had a tendency to overannotate (compared to annotator_A, that is considered the standard for this study), while a statistically significant drop was observed when the annotator_B was an underannotator.

#### 3.1.2. Empirical Work: Typos Correction

Reducing word variability can be helpful for any text mining technique we want to apply. For this reason it was considered appropriate to proceed with techniques to correct typing errors (typos) that can be identified in an automated way.

##### 3.1.2.1. The methods and technologies chosen to solve the problem

The first step of our empirical work was to split the text into words (tokens), deleting the most frequent known words (stopwords), numbers and non-ASCII (American Standard Code for Information Interchange) symbols. The latter were produced in the transition from the systems used in the hospital to the different encoding in DataReg.

We then counted the words frequency in all the available documents, then we considered the area under the power law curve of the words frequency in the whole corpus and finally we assumed that the words occurring in the 80% were correct.

Among the less frequent words, we checked if they were present in an Italian vocabulary^6^ and/or in a medical vocabulary, provided by an external consultant of IOG. The residual words were considered potentially typos. To correct them, it was necessary to identify the right words to which they correspond. To do this, we used a distance metric between strings. First, we considered the distance of Levenshtein (the so-called edit distance) to search, in 80% of the most frequent words, those with distance 1 from potential typos. This means that words that differ for one letter insertion, deletion or substitution from the original are found (45).

However, a very common mistake in typing is the inversion of two adjacent letters that, in the distance of Levenshtein, is considered distance 2. If we take into account also this type of error we could probably confuse some correct words, such as “hypothyroidism” and “hyperthyroidism.” Therefore it was decided to use the distance of Damerau-Levenshtein that also takes into account the inversions between letters.

The words identified were then manually inspected to verify that there were no association errors, such as units of measurement (mmol compared to pmol). The ambiguous associations have been discarded.

In cases where a word was multi-associated (more than one match), it was replaced with the most frequent one. This condition has occurred because the terms have not been normalized to their base form: the wrong words would not have been recognized, while the more similar forms (plural, conjugations) would have been lost, making the identification of typos more difficult.

##### 3.1.2.2. The proposed solution

Using the Damerau-Levenshtein distance, a total of 774 misspelled words were found. Table 1 shows a summary of the first 10 most frequently wrong words (Total errors) with associated the number of variants identified by the algorithm (Number of variants) and the percentage of the ratio between the number of typos and the number of times that the word appears correctly written (Incidence).

**Table 1:** Summary of the most frequent errors identified in the anamnestic summaries of Endocrinology and Rheumatology.

**Correct word**	**Number of variants**	**Total errors**	**Incidence (%)**
Esami	28	147	1.5
Discovery	8	134	13
Somministrazione	6	112	19
Prednisone	9	111	12
Polimialgia	6	106	19
Problematiche	25	106	3.5
Osteoporosi	20	103	1.9
Fratturativa	7	100	11
Terapia	20	98	0.72
Femorale	10	94	6.8

*The first column indicates the correct word, the second indicates the number of variants in which the word was found, the third contains the total of the times in which the word was entered incorrectly, the last contains the percentage of errors compared to the number of times that the same word appears in its correct form*.

##### 3.1.2.3. Critical evaluation and future works

We noticed that the most frequent errors occurred in words of medical jargon rather than in those of common use. This is a potential data quality problem that could affect the medical texts classification. Furthermore, the percentages of wrong words incidence are often quite high and at least not negligible. The words discarded (marked as false positives) during the manual inspection were 3.4% of the total words reported.

Below are reported some limitations of the proposed method.

The number of false positives is quite high, but a possible solution could be to enrich the initially used dictionary to skip the words with units of measurement, abbreviations and acronyms typical of the medical lexicon.

Moreover, in order to fix spelling mistakes in an English document, one can use the Soundex^7^ or the Metaphone^8^ algorithm, which forms equivalence classes based on phonetic heuristics. These approaches would lead to the correction of syntactic errors, and not only typing errors. However, this kind of errors are more common in languages phonologically opaque (such as English), rather than in Italian. The proposed approach has the advantage of being, thus, more language-agnostic.

However, Italian is morphologically more complex than English and allows more flexibility in word order (46). In the Italian language, many ambiguities are observed due to the polysemantic nature of many terms. For example, the past participle of “*affliggere”* (“*affetto”*), widely used in reference to pathologies, could be confused with the name “*affetto”* or with the past participle of the verb “*affettare.”* This is certainly a limitation of our work, which finds different solutions in literature, mainly in the Machine Learning domain. The performances reported in literature are very promising, but often require a large set of data to train, whilst our approach can be considered ready-to-use even in small clinical settings without many possibilities to train computational-intensive models.

Obviously, the suggested procedure does not guarantee to correct all errors, leaving those with a spacing between strings higher than one unaltered. However it is assumed that these cases are less frequent and, therefore, negligible.

### 3.2. Information Extraction

#### 3.2.1. Related Works

**Figure 2:**
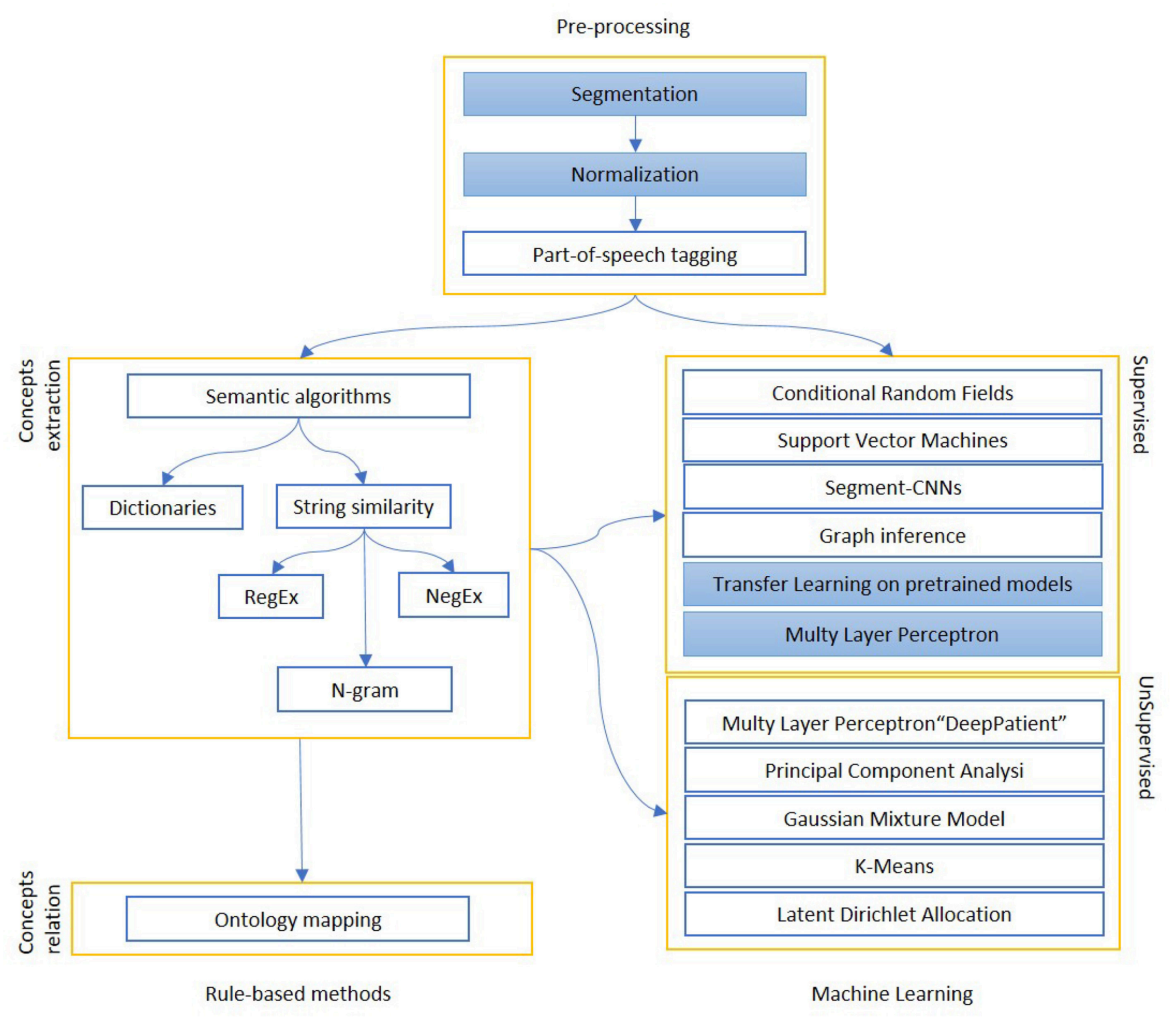
The figure shows the most relevant information extraction techniques available in literature. There is always a first step of pre-processing, where documents are tokenized and differently weighted. One approach is to extract known entities on a pattern-basis, with rule-based methods. These entities can be matched to an existing ontology or used to create one, or intended as a first step for a machine learning algorithm, which can be supervised (mainly classification) or unsupervised (clustering). The colored boxes highlight the experimental approach proposed in this article.

We report in Figure 2 a diagram that presents the most relevant methods related to the topic of “Information extraction” as a summary of the methods present in the literature and which we have reported in more detail below.

About Information Extraction (IE) we found two relevant and recent reviews (17, 18). While the first is focused on radiologic reports, the second is more general. The main finding of Pons et al. (17) study is that NLP could be useful in radiology since radiologists express their diagnosis in free form text that can be converted in structured data only by using NLP techniques. The review by Wang et al. (18) stated that the majority of the analyzed studies (between 2009 and 2016) presented NLP applications based mostly (60%) on a rule-based approach rather than on ML although, in the NLP academic domain, machine learning is considered more challenging. The rule-based approach needs to identify the rules of the named entity from medical texts and the ML method, with good performances in recognizing entities, requires standard annotations training dataset (41).

From these works we extract some relevant research studies. Esuli et al. (47) tried to apply supervised learning methods to extract information from radiology reports (in particular mammography reports). In particular, they proposed two novel approaches to IE: (1) a cascaded two-stage method with clause-level linear-chain conditional random fields (LC-CRFs) taggers followed by token-level LC-CRFs taggers; (2) a model that uses the output of the previous system as input to a traditional token level LC-CRF system. They compared the results of the two ML methods to the annotators tagging: both the systems outperformed in relation to the annotators according to the final values of F1 score. On the other hand, the aim of the study conducted by Li et al. (48) was the implementation of a NLP-based hybrid algorithm for the detection of discrepancies between discharge prescriptions (structured data) and medications in clinical notes (unstructured data). They analyzed clinical notes and prescriptions of 271 patients of Cincinnati Children's Hospital Medical Center. The overall method consisted of 3 processes: a ML algorithm for the detection of medical entities, a rule-based method to link medication names with their attributes and finally a NLP algorithm to match the medications with structured prescriptions. The performances of the processes were assessed by precision, recall and *F*-value. The proposed approach showed good performances in medication entity detection, attribute linkage and medication matching. The method achieved 92.4%/90.7%/91.5% (precision/recall/*F*-value) on identifying matched medications and 71.5%/65.2%/68.2% (precision/recall/*F*-value) on discrepant medications.

Talking once more about radiologist reports, Tan et al. (49) conducted a study where they tried to build a NLP system to correctly identify 26 lumbar spine imaging findings related to LBP (low back pain) on MR (magnetic resonance) and x-ray radiology reports. First, they used NLP to extract information from text using segmentation, normalization, concept identification and negation identification, then they developed two kinds of models, rule-based models and ML models, to identify findings in radiologists' reports. They implemented the rule-based model in Java, looking for keywords, defined as regular expressions (RegEx), and observing if those keywords were denied (NegEx). The ML model was implemented in R (with the caret package), and it used both input functions based on the output of the rule-based model (Regex and NegEx), plus other predictors. In the testing sample, rule-based and ML predictions had comparable average specificity (0.97 and 0.95, respectively). The ML approach had a higher average sensitivity (0.94, compared to 0.83 for rules-based), and a higher overall Area Under the Curve (AUC) (0.98, compared to 0.90 for rules-based).

The use of dictionaries can help to facilitate the extraction of concepts, but often do not contain all the slang words (low recall) or contain ambiguous terms (low precision). The goal of automated coding is to convert free form text (unstructured data) in a code suitable form using for example dictionaries, coding systems, inside-outside-beginning (BIO) notation and concept extraction. A systematic review by Kreimeyer et al. (20) analyzed 86 papers that applied 71 NLP systems to convert unstructured clinical notes into standard terminologies such as UMLS.

Named-entity recognition (NER) is a sub-task of information extraction. It is used to identify medical entities that have specific significance for the treatment (diseases names, symptoms and drug names). Usually 3 parameters are used to evaluate NER: F-score, recall and precision. In the medical field NER methods can be divided into 3 types: the rule-based approach, the dictionary-based approach and the machine learning approach (41). In particular, the dictionaries based approach is a technique that needs medical text annotations and indexing.

The heterogeneity is an intrinsic feature of EHR data. Pivovarov et al. (50) used a large set of heterogeneous data taken from EHR, such as diagnosis codes, laboratory tests, and free-text clinical notes, to build computational models of diseases, based on an unsupervised, generative model (UPhenome). The system, given a large set of EHR observations, learns probabilistic phenotypes. They compared the Latent Dirichlet Allocation (LDA) model with UPhenome. The evaluation metrics they used to assess the coherence of the identified phenotypes with respect to human judgement is NPMI (Normalized Pointwise Mutual Information). They found little correlation between the clinician's judgments and the NPMI of the learned phenotypes. It is possible that the computationally coherent are not actually clinically relevant.

Many studies focused on the analysis of clinical notes structure. They observed that clinical observations are frequently negated in clinical narratives. An example of NLP tool for negative sentences detection is NegEx, that is a simple algorithm. It performs better on simple sentences, with 94.5% of specificity (51). Starting from NegEx, another group (52) of researchers tried to improve the performances of negation detection in clinical texts. They developed a negation algorithm called DEEPEN that decreases the number of incorrect negation assignment in more complex sentences. Compared to NegEx, DEEPEN takes into account the relationship between negation words and concepts achieving a reduction of false positives (i.e., high precision) resulting in an increment of specificity: the precision values are, respectively, 0.91 (NegEx) and 0.96 (DEEPEN) as for the pancreatic concepts. In contrast, the performances are not always improved in terms of recall: it depends on the dataset they used.

It is well known that clinical notes contain information about medical events including medication, diagnosis and adverse drug events. The extraction of medical events and their attributes from unstructured clinical texts is one of the most faced up topic by researchers (53). An original work (27) stated that the implementation of text-based approaches, compared to traditional methods (without NLP techniques), improves significantly the process sensitivity for the identification of medical complications, despite of a small reduction of specificity. Moreover, the use of Natural Language Processing offers a more scalable system than manual abstraction.

Concerning a similar issue, the work by Tvardik et al. (54) analyzed EMRs in order to identify HAI (healthcare-associated infections). Actually, they focused on the implementation of semantic algorithms and expert rules. The results of the automated detection were compared to the reference standard. In particular, the overall pipeline is made up of 3 processing steps: the first is a terminological normalization that includes a concept extraction tool (ECMT v2). The second stage involves a semantic analysis with the help of the platform called MediParser. Finally, the pipeline includes an expert knowledge processing that performs temporal labeling and section classification. The method showed good performances, with an average accuracy of 83.8%, a specificity of 84.2% and an overall sensitivity of 83.9%. Finally, it was found that inaccurate temporal labeling was the most frequent cause of classification errors. On the contrary, a study conducted by Branch-Elliman et al. (55) demonstrated poor performances for the detection of the real-time CAUTI (Catheter-Associated Urinary Tract Infection) with a NLP algorithm (data extraction and processing) compared to standard surveillance results (manual). The problem was probably affected by language patterns that are local to a specific setting where the model has been trained. On the other hand, the implemented NLP system was most useful for the identification of clinical variables.

Xu et al. (56) mined concepts, classified assertions and identified relations from medical records to help physicians in clinical decision making. The overall system consisted of many steps. First, the sentences were pre-processed so that NLP tools could be applied. Second, the authors used SharpNLP to mark noun phrases and adjective phrases. After that, a concept-extractor model, based on conditional random fields (CRF) method and on BIO notation, was applied to divide the 3 main concepts: treatment, problem and test. Then, the medical problems were associated with an assertion class: present, absent, possible, conditional, hypothetical and not associated. Five classifiers were implemented to classify the assertions. The results suggested that a rule-based classifier, implemented by manually constructing a large and comprehensive dictionary, showed the best performance evaluated with F-measure: 0.85 for concept extraction, 0.93 for classification and 0.73 for relation identification that are good results compared with the state-of-the-art.

Jackson et al. (57) investigated the feasibility of an automated method to extract a broad range of SMI (Severe Mental Illness) symptoms from EMRs. They used TextHunter that is a NLP tool for the creation of training data and for the construction of concept extraction ML models. It is a flexible SVM based algorithm to extract concepts. The simple annotation interface enables a rapid manual annotation process to create training data. The implemented model, based on SVM method, extracted data for 46 symptoms with a median F-score of 0.88. This study did not attempt to resolve temporal aspects for predictive modeling.

A study by Carrell et al. (58) tries to use NLP to monitor the therapies that use opioid. They assess that accurate and scalable surveillance methods are critical to understand widespread problems associated with misuse and abuse of prescription opioids and for implementing effective prevention and control measures. At the end of the study they concluded that there are certain information retrieval tasks for which neither a fully-automated NLP system nor traditional manual review are initially feasible, but which can be accomplished using a hybrid strategy of NLP-assisted manual review.

Zeng et al. (59) developed an open-source, reusable, component-based NLP system called HITEx (Health Information Text Extraction), based on open-source NLP framework (GATE). The overall pipeline consists of several components: starting from a section splitter of the medical records the process continues with a sentence splitter and tokenizer. The next step includes the POS (part-of-speech) tagger and the mapping of the strings of text to UMLS (Unified Medical Language System) concepts. The last steps of the process include Negation finder, N-gram tool, Classifier and Regular expression-based concept finder. Their goal was the extraction of principal diagnosis, co-morbidity and smoking status on 150 discharge summaries. The results, compared to a human-created gold standard, showed that the overall accuracy was in the range of 70–90%, generally comparable to other similar NLP systems.

Two groups of researchers (9, 60) proposed a different approach that consists in reusing existing NLP applications and adapting them to new challenging tasks. The aim of the study by Khalifa et al. was to identify cardiovascular risk factors in narrative clinical records. They defined 8 categories of information that represent risk factors for heart disease. The researchers presented the results achieved by implementing 2 existing tools based on the Apache UIMA (unstructured information management architecture): Text Analysis and Knowledge Extraction System (cTAKES) and Textractor (61). The cTAKES is an open source modular system of pipelined components combining rule-based and machine learning techniques aiming at information extraction from the clinical narrative (62). It can be used to preprocess clinical text and to classify the smoking status. The identification of chronic disease mentions is carried out by the dictionary-based lookup component of Textractor. Eight quality measures were extracted with high performance, achieving F measures 0.90 at each site.

Structuring medical records is often carried out through the construction of *ad hoc* ontologies for the individual departments, in a restricted domain, with a strong interaction between doctors and data experts. There is a trade-off between completeness, quality and completion time for any type of standard and, necessarily, of the three dimensions it is possible to obtain at the same time only two. The medical domain has over one hundred different standards, which over the years have tried to cover all the knowledge of the sector, but without ever being able to provide a global and satisfying vision. A systematic review by Vuokko et al. (19) states that the most studied structuring methods aimed to convert unstructured data into UMLS, International Classification of Diseases (ICD) and Systematized Nomenclature of Medicine (SNOMED) codes. As a matter of fact, many groups of researchers (63–66) tried to develop a method, based on NLP techniques, that automatically assigns medical codes to clinical concepts, because manual coding can be noisy and not very fast. For example, Perotte et al. (63) built an automated NLP application based on ICD9 codes. First, they studied ICD9 diagnoses codes and they analyzed many discharge summaries. The results showed that a hierarchical classification behaves better than a flat classification (that considers the codes as independents) with F measures of 39.5% and 27.6%, respectively. The goal of another research group (64) was to improve the performances of automated encoding. They used a supervised learning approach to assign diagnosis codes (ICD9) to a large EMRs dataset. They experimented three base classifiers: Support Vector Machines (SVMs), Logistic Regression (LR), and Multinomial Naive Bayes (MNB). Moreover, the results of Baumel's study on ICD9 codes showed that careful tokenization of the input texts and hierarchical segmentation of the original document allow to yield the most promising results (66).

We move from the extraction of single concepts, to the extraction of durations and frequencies of the therapies, from the extraction of temporal events (TE) to the extraction of relationships. Many groups of researchers (67–70) studied how to automate these specific processes. In particular, Kovačević et al. (67) developed a method that automatically recognizes TE and assigns 3 attributes: value (using ISO representation), type (Time, Date, Duration, or Frequency) and modifier associated. First of all, the narratives were pre-processed with a rule and dictionary-based algorithm. Then, TE were extracted and normalized, as before said. The results showed a good value of F-score (90.08%) with a recall of 91.54% for the TE identification. They considered 1820 temporal expressions in 120 clinical narratives. Concerning temporal relation identification from clinical notes, Nikfarjam et al. (68) realized a system that discovers patterns in sentences to extract temporal links. They showed that the combination of graph inference and ML-based classification is a good method to identify the relationships between TE. The overall performance of the system was assessed in terms of F-measure (0.64), precision (0.71), and recall (0.58). Moreover, this technique is domain-independent, so that it can be applied in other contexts.

Research in the field of structuring EHRs is very active (1), especially with Deep Learning techniques. In recent years Deep Learning has started to be widely used in the Machine Learning domain thanks to his power to explore data deeply. For example, Neural Networks (NNs) are excellent in extracting relevant patterns from sequence data. In a study involving Mount Sinai data warehouse patients (26) wanted to demonstrate the importance of feature selection and data representation to obtain the best possible predictive and classification performances. They proposed an unsupervised feature learning (called “DeepPatient”) to automatically identify patterns and dependencies in the data by a MLP (Multy Layer Perceptron). Then they evaluated the system using 76,214 patients of the data warehouse in two applicative clinical tasks: disease classification and patient disease tagging. In both tasks the DeepPatient technique showed better performances in terms of AUC (0.77), accuracy (0.93), and F-Score (0.18) compared to more traditional approaches such as PCA (Principal Component Analysis), GMM (Gaussian Mixture Model) and K-Means. Actually, deep learning and in particular CNNs are able to detect deep relations between data. For example, Luo et al. (71) proposed a work in which they use Segment-CNNs (Seg-CNNs that is a variation of CNNs) to classify the relations from clinical notes. Indeed, there are some studies that assessed that it is important to not only identify the conceptual entities but also the relationship between these concepts (72–75). The research uses the i2b2/VA relation classification challenge dataset and they showed that Seg-CNNs achieved state-of-the-art performances on relation classification without previous manual feature engineering.

#### 3.2.2. Empirical Work: Structuring Texts

This section deals with the structuring of Hospital Discharge Registers (HDR) of San Siro, an hospital from the same group, present in the database in the form of blobs, following the example of those of Galeazzi, coming from the specialty of hip and knee.

##### 3.2.2.1. The methods and technologies chosen to solve the problem

Since the texts were not labeled in the San Siro systems, we trained a model on the IOG data. Each section was split into sentences and assigned to one of the previously identified parts. This partition was based on the available punctuation. More fine and complex systems (able to recognize the parts of the speech and the syntactic structure of the sentence) were not used in order to speed up the process. The number of sentences resulting for each section are:

- Anamnestic summary (8,293 sentences)
- Diagnosis specific treatments (3,098 phrases)
- Pharmacological therapy (3,553 phrases)
- Rehabilitative program (4,778 sentences)
- Others (48,232 sentences)

We performed the usual tokenization operations and the text cleaning from stopwords and errors described in the previously. The sentences were then transformed into vectors using the Doc2Vec (76) technique. The available texts have been split into train and test set (80:20 ratio). The classification was performed considering a class of interest vs. all the others (one vs. all). A logistic classification was applied, using the Glmnet package in R^9^.

Initially, we considered the classes with the actual proportions (unbalanced), then we implemented a classification with balanced training set. In the latter case, the class of interest was represented at least at 40% in the training set, while in the test set the proportions were still the original ones.

The model training was performed with 10-fold cross-validation on the training set, in order to reduce the variance. In the glmnet R package cross-validation, stratified random sampling is applied, to balance the distribution of target classes between the splits.

##### 3.2.2.2. Methods to evaluate and compare alternative solutions

To compare the solutions we assessed the performance of the two models in terms of Area Under the receiver operating characteristic (ROC) curve (AUROC).

##### 3.2.2.3. The proposed solution

**Figure 3:**
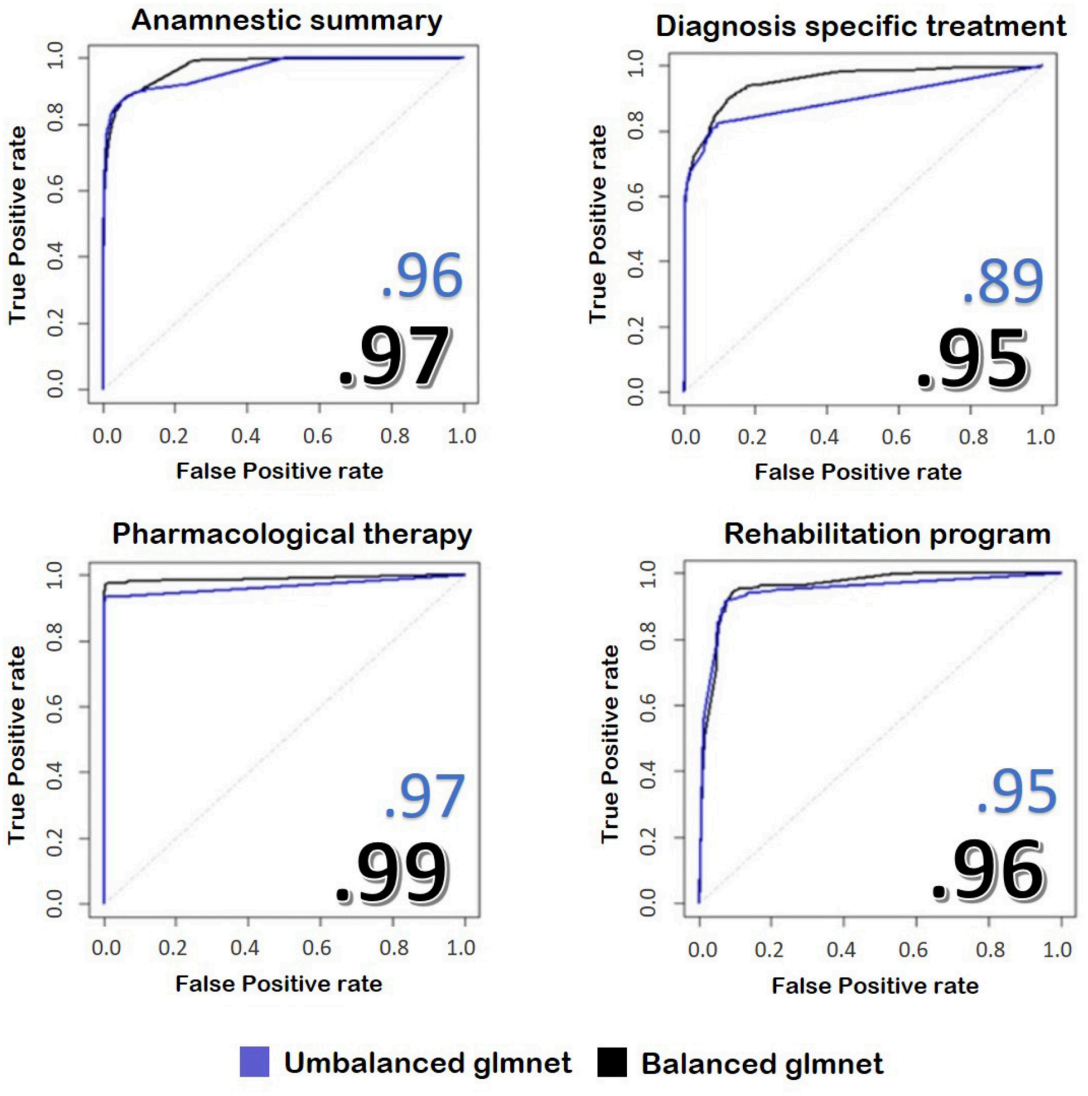
The image represents the ROC curves for the classification of four fields of the discharge letters of hip and knee. In blue, we see the results taken from an unbalanced training set, while in black we see the application of the same model trained on balanced train data. The numbers reported on the figures show the performances achieved by the two implemented models.

Below we report the results of only some representative categories. Figure 3 shows the ROC curves and the performances achieved by the two models.

##### 3.2.2.4. Critical evaluation and future works

We found that the model with balanced classes performed better in predicting the class of the sentences compared to the model with the original proportions, despite it was trained on less data. Our model reaches values similar to other works, such as Tan et al. (49), but with the advantage of skipping the demanding pre-processing rule-based part performed by these authors.

A limitation of the proposed solution is that it was not possible to test it extensively on the data from the San Siro information systems. Indeed, we have been provided with only few copies of HDRs, which do not make possible a significant statistic for the evaluation of the actual classification target. In fact, the reported performances refer to tests on the documents coming from IOG. However, we believed that the lexicon and the writing style of San Siro's documents may be quite similar, but domain adaptation (77) could become a serious issue if different guidelines and templates are in use in different hospital settings.

#### 3.2.3. Empirical Work: Named-Entity Recognition

In this paragraph we will discuss about how to train a model in order to extract interesting entities, which is useful for the structuring of texts and their tagging.

##### 3.2.3.1. The methods and technologies chosen to solve the problem

Named Entity Recognition refers to the application of pre-trained models for the extraction of concepts. Several software were considered for this activity, including Stanford CoreNLP (78), Tint (79), and SpaCy^10^. The common problem is the difficulty in extracting precisely the most interesting entities for the case in question, such as medical jargon. To overcome this problem, it was decided to use a transfer learning algorithm, so we chose SpaCy. Unlike Stanford CoreNLP and Tint, that use Conditional Random Fields, SpaCy is based on a neural network model with Attention^11^. The model allows to replace the last training layer with a customized one and, with a few significant examples, it allows to learn new entities, such as drugs, quantities or diseases. For this reason, we tagged with the new entities 400 texts for training and 50 for the test drawn from the anamnestic summaries of Endocrinology and Rheumatology. The tagging was done with the Brat software (80), with the BILUO scheme^12^.

To avoid that SpaCy, after the new training step, would forget the previously learned tags (i.e., catastrophic forgetting), it is recommended to continue the training with the addition of new tags, together with those previously identified by the algorithm.

##### 3.2.3.2. Methods to evaluate and compare alternative solutions

Once we trained the new model, the confusion matrix was extracted for the new entities, considering them individually, compared to the total amount of the extracted ones.

Since we considered that the new extracted tags were more important than those previously proposed, we made an attempt to train with voluntary catastrophic forgetting, in order to improve performance on the entities of interest. The training without catastrophic forgetting was done with 250 iterations, while the one with the catastrophic forgetting with only 100, because it converged faster.

##### 3.2.3.3. The proposed solution

First, we extracted the entities by SpaCy in its basic version from an anamnestic summary of Endocrinology and Rheumatology.

We report below the confusion matrices created by the model trained on all the entities (Table 2A) and the one with only three entities of interest (Table 2B). “All” indicates the set of all entities other than Drug, Illness and Quantity, while “Null” indicates untagged entities. The chosen entities are reported in the center of the matrix.

**Table 2:** Confusion matrices resulting from transfer learning.

**A**					
**Predicted**					
**True value**	**All**	**Disease**	**Drug**	**Quantity**	**Null**
All	1251	10	17	2	192
Disease	7	143	0	0	47
Drug	16	0	128	0	7
Quantity	0	0	0	56	36
Null	137	33	51	10	8
**B**					
**Predicted**					
**True value**	**All**	**Disease**	**Drug**	**Quantity**	**Null**
All	0	16	25	2	1492
Disease	0	149	0	0	48
Drug	0	2	137	0	12
Quantity	0	0	0	68	24
Null	0	38	54	18	8

*The table above is referred to the application of transfer Learning on all entities (without forgetting), while the table below is referred to the application of transfer Learning on the entities of interest only (with forgetting)*.

Table 3 shows the results obtained divided by class. We evaluated sensitivity, specificity and accuracy considering only the three tags of interest. It is therefore intended that the “Null” and the “All” are considered the same, because the “All” are not considered relevant to evaluate the model. The results showed an accuracy of 0.88 in the case of the training without catastrophic forgetting and a performance of 0.89 in the other case.

**Table 3:** Sensitivity, Specificity and Accuracy of the models obtained after the transfer learning on the anamnestic summaries of Endocrinology and Rheumatology, without forgetting (NF) and with forgetting (F), considering the three classes of interest with respect to all the others, classes not interesting for “non-classes.”

	**Disease**		**Drug**		**Quantity**		**Other**	
	**NF**	**F**	**NF**	**F**	**NF**	**F**	**NF**	**F**
Sensitivity	0.72	0.76	0.85	0.91	0.61	0.74	0.93	0.90
Specificity	0.97	0.97	0.96	0.95	0.99	0.99	0.74	0.81
Accuracy	0.95	0.94	0.95	0.95	0.97	0.98	0.89	0.88

In the case we would consider that even the previously tagged entities were important, the situation would change drastically: the total accuracy without catastrophic forgetting is 0.74, but with catastrophic forgetting it becomes 0.18. Table 4 shows the details divided by class, focusing on the “lost” tags: anything that is not the three entities is classified as one of them or it is not classified at all.

**Table 4:** Sensitivity, Specificity and Accuracy of the models obtained after the transfer learning on the anamnestic summaries of Endocrinology and Rheumatology, without forgetting (NF) and with forgetting (F), considering the three classes of interest with respect to all the others and “Not classes.”

	**Disease**		**Drug**		**Quantity**		**All**		**Null**	
	**NF**	**F**	**NF**	**F**	**NF**	**F**	**NF**	**F**	**NF**	**F**
Sensitivity	0.73	0.76	0.85	0.91	0.60	0.74	0.85	0	0.03	0.07
Specificity	0.97	0.79	0.95	0.74	0.99	0.94	0.68	1	0.76	0.19
Accuracy	0.94	0.78	0.95	0.80	0.97	0.89	0.81	0.20	0.75	0.18

##### 3.2.3.4. Critical evaluation and future works

Considering the proposed solution, it is clear how it was possible to refine the extracted entities compared to the model without transfer learning. The main problem of the pre-trained model application is the great difference between the essential style of the medical texts, compared to the one on which SpaCy was trained for the Italian language, which is a corpus of thousands of editions of the newspaper “Gazzetta dell'Alto Adige.” We have seen how the training based on the selected entities led to a total forgetting of the labels normally assigned by the model. In general, this is not desidered, even though many of the previously recognized entities did not seem appropriate. In any case, the highlighted model, although it is not very powerful it enables transfer learning, which instead produces promising results.

It is clear that more complex model in literature may perform better, but we stress once again the relatively small effort to achieve State of the Art-level performances without big annotated datasets. In comparison to rule-base methods, we propose a model which can be pre-trained to completely different datasets, overcoming the difficulty of the scarcity of annotated datasets.

Furthermore, there are several issues for future research that this work leaves open. First of all, a fundamental passage on which it will be necessary to dwell in the case of the concept extraction will be the management of negatives. There are algorithms and many literature contributions on this specific field (52), but it is a challenging task, especially in languages like Italian, which admits various constructions for sentences and the use of double negatives. In addition, it may be helpful to consider, in addition to the single words and their combinations in bigrammes, also the N-grams, in the sense of groups of letters. This is particularly useful in identifying similarities between otherwise different terms, such as in the case of prefixes or suffixes (for example, “farmacoterapia” vs. “chemioterapia”).

The next step should be to obtain a finer structure of patients' data: once the entities are extracted, they should be placed in relation to each other, associating the drugs, quantities and diseases correctly. The structuring could continue in the creation of databases containing the surgery data of the patients, on which it may be easy to perform queries. The most suitable format for such varied data seems to be a document database, like MongoDB, which favors the patient's centrality and contains the highly differentiated data found in the texts analyzed.

### 3.3. Sentiment Analysis and Predictive Models

#### 3.3.1. Related Works

**Figure 4:**
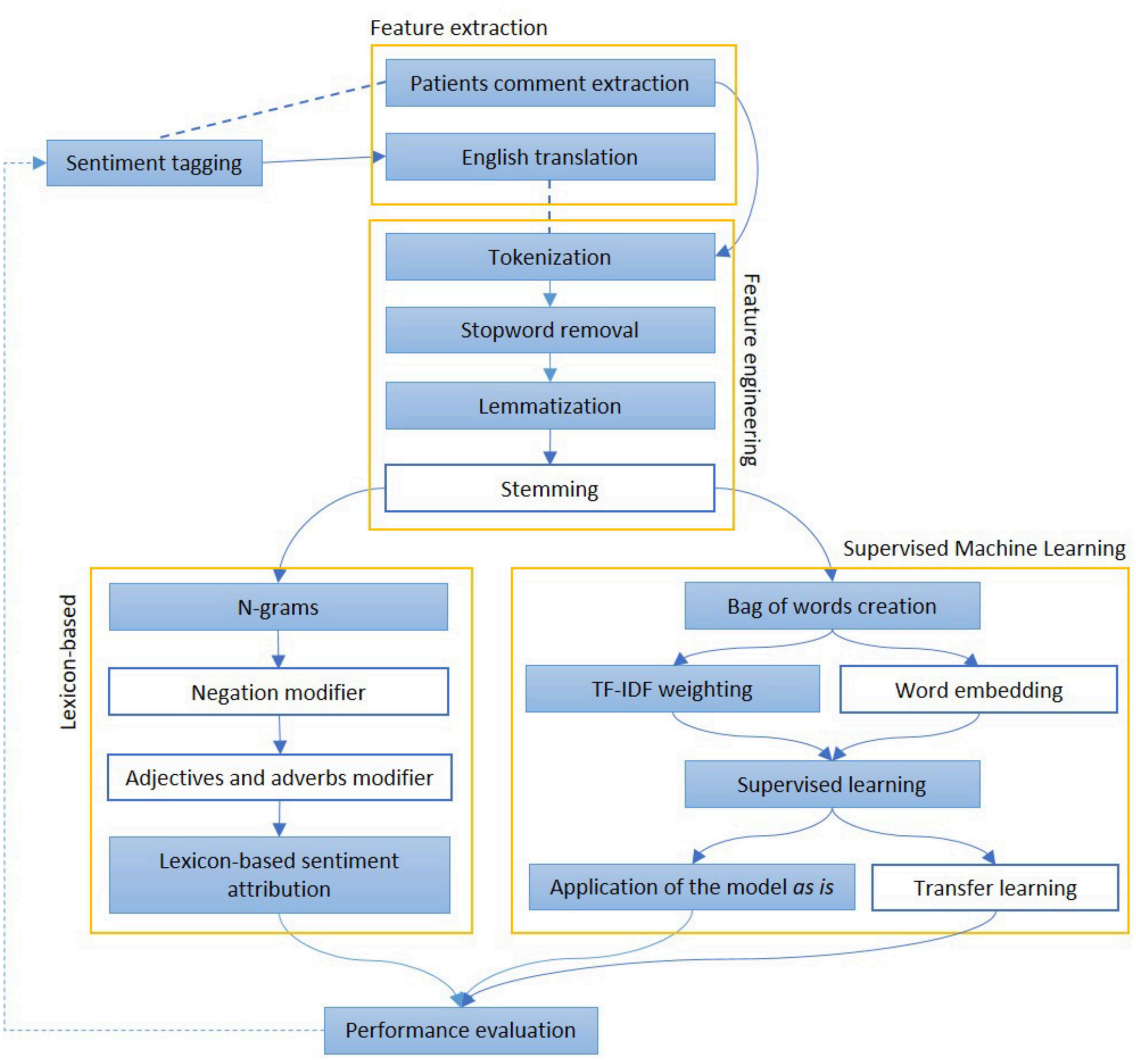
The figure represents the most common steps to perform and evaluate sentiment analysis. After a pre-processing phase, when documents are divided into words and normalized, Lexicon-based and Machine Learning-based approaches are described. In the first group of methods, each word may have a polarity, which can be modified by the surroundings. In the second group, the whole sentence is assigned with a sentiment, on the basis of other sentences, adapted from a similar domain The colored boxes highlight the experimental approach proposed in this article.

**Figure 5:**
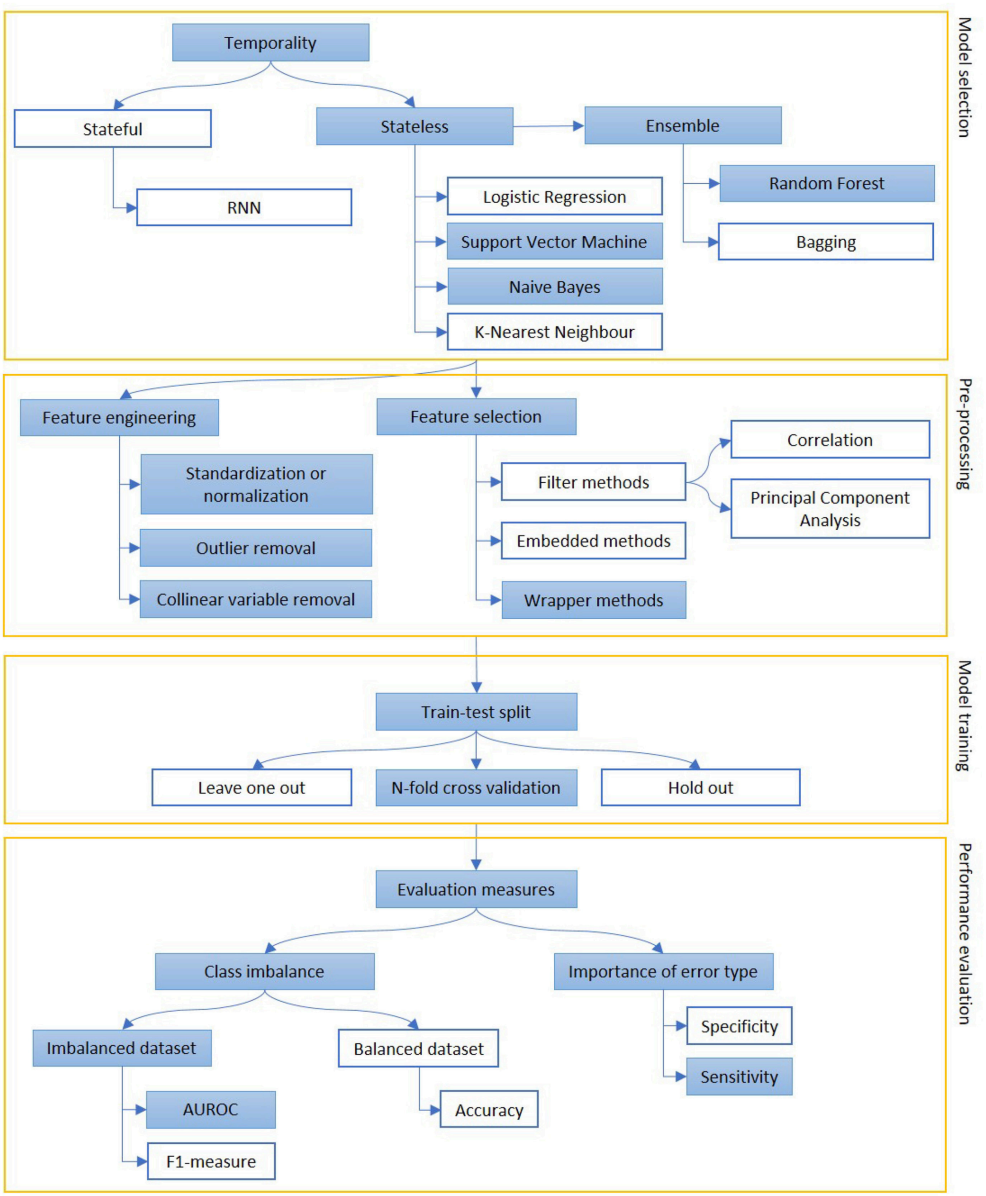
The figure represents the process of creation of a predictive model, mainly focusing on classification. Models can be selected on some prior considerations about the value of temporal order of information (stateful or stateless) and to reduce variance when data availability is scarce (ensemble methods). The extracted features are engineered and selected and, then, the model is trained on a subpart of the dataset. Performance are evaluated, depending on the goal of the model (sensitivity vs. specificity) and the target class representation in the dataset (accuracy vs. AUROC or F1-measure). The colored boxes highlight the experimental approach proposed in this article.

We report in Figure 4 a diagram that presents the most relevant methods related to the topic of “sentiment analysis” and in Figure 5 those related to “predictive models” as a summary of the methods present in the literature and which we have reported in more detail below.

Sentiment analysis is used to extract information from clinical texts or to judge the impact of a medical condition on patients. The development of this topic can be used as an additional feature to predict the patients' health status. This is why this concept can be related with the implementation of predictive models.

Many reported studies showed that predictive models were usually applied independently from sentiment analysis in clinical narrative applications. For example, Dagliati et al. (81) used different classification models applied to EHR data to predict diabetes complications such as retinopathy, nephropathy, neuropathy at 3, 5, and 7 years from the first visit. The performances of the models were evaluated with a leave one out validation strategy. The study revealed that the Logistic Regression used for a 3 years prevision is the best model choice to be translated into clinical practice, compared to NB, SVM and Random Forest (RF). In particular, the values of the AUC for the retinopathy prediction are 0.75, 0.61, 0.48, and 0.51, respectively, for LR, NB, SVM, and RF.

Another application of predictive methods on clinical texts is developed by Choi et al. (82) that attempted to predict the onset of HF (Heart Failure) using longitudinal structured patient data such as diagnosis, medication, and procedure codes. Temporality of HF onset is fundamental for the research. In actual fact, the study highlighted the power of RNNs (Recurrent Neural Networks) to take into account the time variable to predict future events compared to traditional machine learning models. The results showed that the AUC for the RNN model was 0.78, compared to AUCs for LR (0.75), multilayer perceptron (MLP) with 1 hidden layer (0.77), SVM (0.74), and K-nearest neighbor (KNN) (0.73).

The aim of the study conducted by Agarwal et al. (83) was to predict the readmissions of chronic obstructive pulmonary disease (COPD) patients analyzing clinical notes. The United States health system penalizes excessive readmissions hospitals for excessive 30-day COPD readmissions. The data used to test this system consist of 1,248 clinical notes from COPD patients over the period of 5 years, that have been labeled with the note “readmission,” “not readmission” by annotators. They applied four classification methods (kNN, SVM, NB, and RF) and they evaluated each model calculating the AUROC and model creation time (the average of 10 times). Actually, the application of NB model resulted in a better performance, with AUROC equal to 0.69 while maintaining fast computational times. Accuracy in this case can be misleading because of the unbalance of the two classes.

As before said, a challenging task can be the combination of sentiment analysis and predictive models. Recent research by Van Le et al. (84) rated presence or absence and frequency of words in a forensic EHR dataset, comparing four reference dictionaries to predict the risk of violence in psychiatry patients. They tested 7 different machine learning algorithms (Bagging, J48, Jrip, LMT (Logistic Model Trees), Logistic Regression, Linear Regression and SVM) combined with all the dictionaries to identify the best method. SVM and LMT in conjunction with sentiment dictionary showed a better accuracy (respectively 0.74 and 0.75) of risk prediction compared to the others (from 0.64 to 0.70).

The group of Sabra et al. (85) proposed a Semantic Extractor (SE) to identify hidden risk factors in clinical notes and a Sentimental Analyzer (SA) to assess the severity levels associated with the risk factors and finally make a diagnosis. Their purpose was to implement an open resource in order to be applied for many diseases. In particular, this study aim was to predict venous thromboembolism (VTE) analyzing semantic and sentiment in patients' clinical notes. Their sentimental analyzer finds the correct sense of an adjective or an adverb, then it labels it with either increasing or decreasing criticality. They evaluate 120 clinical narratives, of which 62 are labeled as positive for VTE, with three metrics of evaluation: Precision, Recall and F1-measure that, respectively, correspond to 81% for the SE and to 70%, 60%, 50% for the SA.

McCoy et al. (86) performed sentiment analysis on hospital discharges of more than 17,000 patients. Their aim was to identify the correlation between sentiment in clinical notes and the risk of hospital readmission. In particular, they used Pattern, an open source implementation of lexical opinion mining developed at the University of Antwerp. In brief, this method depends on matching words and phrases to an included lexicon of nearly 3,000 words annotated for polarity, subjectivity, intensity and negation. In this approach unrecognized words (those not included in the lexicon) are ignored. Results showed that greater positive sentiment predicted reduced hospital readmission. Moreover, the automated characterization, in terms of sentiment, demonstrated differences between socio-demographic groups.

#### 3.3.2. Empirical Work: Sentiment Analysis

In this section we want to analyze comments on specific topics, with the aim of creating alerts based on potentially negative sentiment.

##### 3.3.2.1. The methods and technologies chosen to solve the problem

In order to extract the sentiment from the comments of patients regarding their health and to create alerts on possible alarming conditions, it is first necessary to discriminate the topics of the comments.

Actually, they can be divided into two macro categories: on the one hand, those relating to the service, identifiable as customer satisfaction; on the other hand, the Patient-Reported Outcomes (“outcomes”). Going deeper, the comments regarding satisfaction can be distinguished between comments on the hospital service (such as cleaning and schedules: “hospitality”) and those on the clinical side (such as missing information, displaced examinations and nurses' availability: “nursing”).

In order to establish a *ground truth* three different people labeled the sentiment of the comments (positive, negative and neutral) and the classes to which they belong (hospitality, nursing and outcome). The eventuality of “no comments” was not taken into account.

The final class has been assigned considering the mode, after a verification of a high enough Krippendorff alpha (0.69).

Once divided by type, we applied two different techniques to verify the sentiment. First we counted negative and positive words by using the Vader library^13^. In addition to the single word, the library also considers the bigrams, that could change the meaning of some words such as in the case of “not improved.” Second, we used a corpus of pre-labeled tweets based on sentiment^14^. This means that we had a general corpus, from which it was possible to extract identifiable patterns even within the patient's comments.

In this second case, we created Bag of Words weighted with TF-IDF, after the usual tokenization, the removal of the stopwords and, in this case, also the lemmatization. We used neural networks and we chose the following hyperparameters for the training: a maximum number of 1,000 iterations and a step of 0.001. In both cases, we considered a binary classification, in which the class of interest consisted of the negative comments, compared to all the others (positive and neutral).

Since the library and the labeled data were in English, patients' comments were previously translated using the Google Translate API (Application Programming Interface).

##### 3.3.2.2. Methods to evaluate and compare alternative solutions

We evaluated the two proposed solutions compared to the *ground truth* of the manually labeled comments. In both cases, all the available comments were used as a test, because in the first case the model was already provided by the library, while in the second case the training set consisted of tweets. Since our aim was to use existing models, no training or tuning was done to establish the optimal thresholds.

The overall performance was assessed in terms of accuracy, sensitivity and specificity.

##### 3.3.2.3. The proposed solution

**Figure 6:**
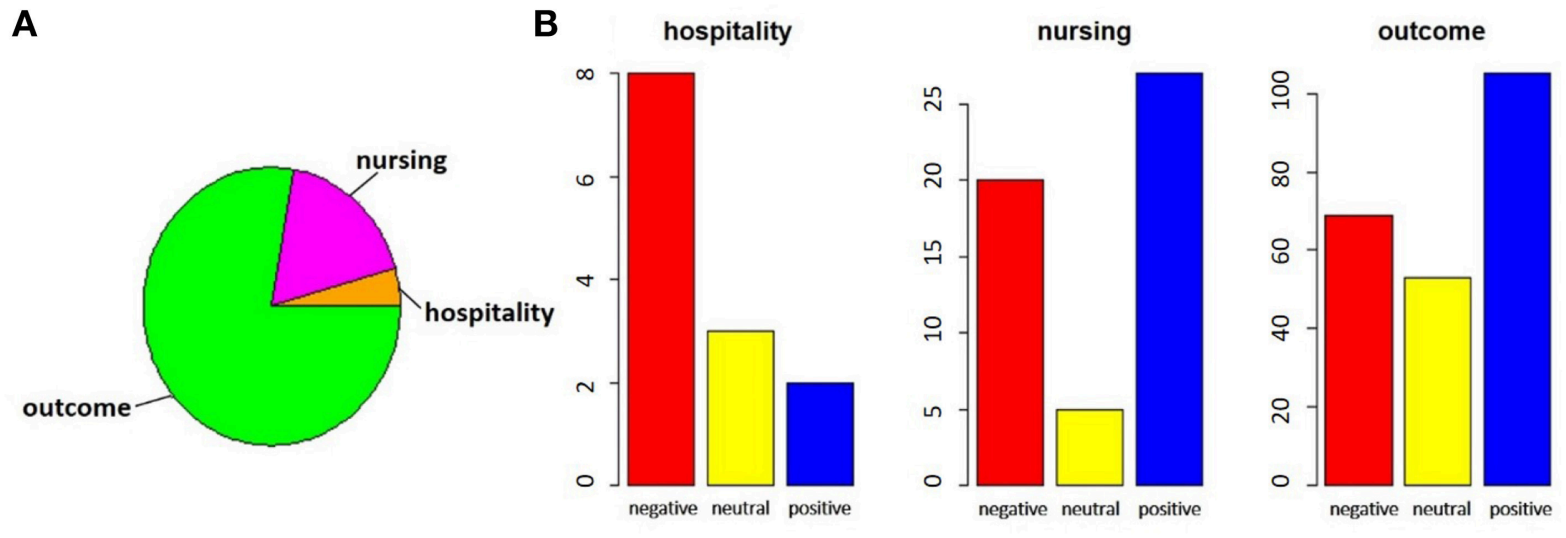
In the pie chart **(A)**, we report the proportions of comments referring to satisfaction, divided between hospitality theme (orange) and nursing theme (magenta), with respect to those related to outcomes (green). In bar charts **(B)**, for each of the categories identified, the number of negative (red), neutral (yellow) or positive (blue) comments is highlighted.

The exploratory analysis on the distribution of the assigned tags is shown. Figure 6 shows the distribution of the assigned labels, considering the category (Figure 6A) and the sentiment (Figure 6B).

By performing a proportion test between the total of the satisfaction comments and the outcomes comments, we noted that there is no significant difference between the two parts (χ^2^ = 5.424, *p*-value = 0.066). The same test on the two subparts of the comments on satisfaction, however, showed that the imbalance toward negative comments for hospitality was significant, while the nursing service collected more positive responses (χ^2^ = 10.123, *p*-value = 0.006).

**Figure 7:**
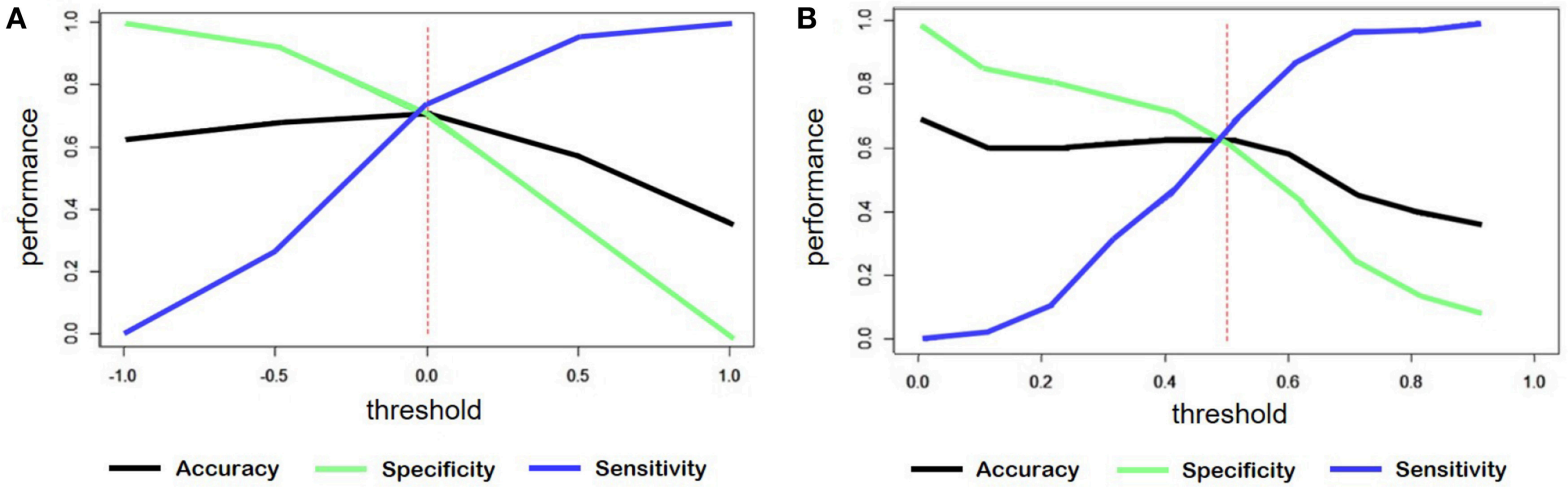
**(A)** Trend of accuracy, specificity and sensitivity according to threshold variations of sentiment predicted for the model created by word counts. **(B)** Trend of accuracy, specificity and sensitivity according to threshold variations of sentiment predicted for the model created through training on tweets.

For the classification of sentiment words counting, the output was a continuous variable ranged from -1 to +1. We considered the outputs less than zero as negative comments. Figure 7A shows the accuracy, sensitivity and specificity trend depending on the threshold.

In the case of the classification starting from tweets, the threshold was set to 0.5, since the predicted variable had a range from 0 to 1. Figure 7B shows, also in this case, the trend of the main evaluation parameters depending on the threshold.

Table 5 shows the correlation values between the predicted variable and the labeled sentiment and also the specific values of accuracy, sensitivity and specificity obtained with these models.

**Table 5:** Comparison of the main performance parameters of the two proposed models.

	**Model 1 (counts)**	**Model 2 (tweet)**
Correlation	0.58	0.19
Accuracy	0.72	0.63
Specificity	0.75	0.61
Sensitivity	0.71	0.66

##### 3.3.2.4. Critical evaluation and future works

Considering all the parameters evaluated, the winning model was the one based on the counts of words provided with sentiment. All three values were above 70%, allowing the creation of alerts on potential health risks without creating too many false alarms (good specificity), or neglecting too many potential risks (good sensitivity).

The model based on tweets was penalized for different reasons. First, the language is much more free and slang. Second, the training set presented only positive and negative classes leaving out the neutrals. The same model, tested just on positive and negative comments, presented an accuracy of 85%, but it would not be applicable to real cases, where there are also comments with neutral sentiment. Therefore, in order to improve performance, it would be useful to identify a training set suitable to the need.

A limitation for both the models proposed was the need to translate the original texts into English. As a future work, the impact of translation must be assessed. Specific vocabularies for Italian will be evaluated, or tweets (or reviews or other) already labeled in our language and, possibly, also including the neutral label.

In addition, both the approaches consider common words polarity only, and never consider the “medical polarity”. In other words, they would not rate a cancer-related drug worse than a influence-related one. The creation of a similar dictionary would be welcome in this field.

#### 3.3.3. Empirical Work: Predictive Models

In this section we can see how, starting from structured data, it is possible to move on to more advanced analyses, such as the prediction of some significant variables.

##### 3.3.3.1. The methods and technologies chosen to solve the problem

Considering the structured data collected from 2013 to the present-day of Hip and Knee prosthetics and from 2015 to today of Spinal surgery, we developed predictive models on the evolution of pathologies. The target variables to predict were the scores of the forms completed by the patients, the so-called Patient-Reported Outcome Measures (PROMs), considered in a temporal step immediately following the surgery.

**Figure 8:**
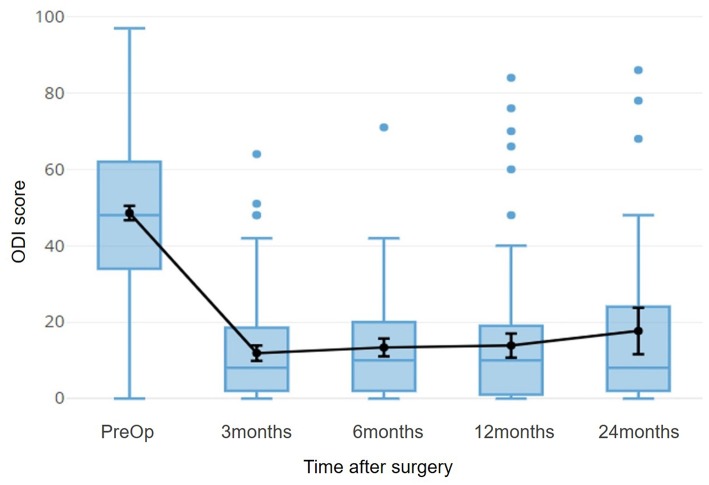
The time course of the ODI score for patients with herniated disc is shown. This index refers to a disability, so 0 indicates the optimal condition, 100 indicates total disability. The black line superimposed on the boxplots refers to the average of the scores, with a 95% confidence interval.

For the part of Spinal surgery, patients of herniated discs were considered, both because they are a fairly large homogeneous population, and because the post-intervention improvement is rapid enough to allow stabilization already in 3 months (Figure 8).

For this part we used the Weka software (87), both for the preparation of the attributes and for the actual training.

The Key Performance Indicator (KPI) that we wanted to predict was the improvement of the Oswestry Disability Index (ODI) 3 months after the surgery, compared to the pre-operative condition. We split patients in improved and not improved, based on the minimum clinically significant difference threshold, indicated as a delta of at least 11.5 points (88). Based on this splitting, there were 42 non-improved patients and 189 improved patients (18% non-improved and 82% improved).

The available data for the prediction come from the scores of the other pre-operative questionnaires filled by the patients (36-items Short Form, Fear-Avoidance Belief Questionnaire, Core Outcome Measure Index), as well as the answers given to a surgical questionnaire, the surgery forms [Spine Tango (89)]. The latter contains all the most important information on the surgical procedure, on the techniques used, on the comorbidity, on the details of the disease. In addition, some personal details were included, such as the gender of the patient, the age at the time of surgery or the BMI (Body Mass Index), but also other variables, such as the duration of the surgery or the month in which it was performed. All the checkbox answers were coded with one-hot technique, while the radio buttons were considered as categorical variables.

The dataset was then split into training and test set in a 75:25 ratio, either randomly, or trying to balance the training set through undersampling, keeping the original proportions only in the test set.

After that, we standardized and normalized the continuous values of the questionnaire scores and we deleted the outliers. This step was applied only after the split between training and test set to avoid the Data Leakage (90) phenomenon: the involuntary introduction in the training set of part of the test set information, which would otherwise have been taken into account in the calculation of minima and maxima, averages and standard deviations for normalizations and standardizations.

For the selection of attributes, we first evaluated collinearity, deleting those with correlation above 0.95. Then, a feature selection method based on decision trees was applied, using Weka's WrapperSubsetEval.

We considered appropriate to manage missing data through imputation techniques due to the lack of data. The percentage of missing data was below 5%, but the difficulty in keeping patients in follow-up and the training set undersampling made every single record valuable. The chosen imputation technique was the kNN, with *K* = 1. After the missing values imputation, a manual inspection of the surgical report allowed us to assess that the values entered were correct.

We made various attempts using different models, including RF, SVM (SMO in Weka), and NB. We performed different kind of features manipulations: the continuous attributes were kept the same or discretised (e.g., in age groups) while the categorical ones (radio button) have been kept the same or replaced by dummy variables (also to evaluate the effect of sparsity). Due to data quantity problems, we chose the hyperparameters of each model with cross-validation directly on the training set without using a tuning set. For the Support Vector Machines we used a polynomial kernel, for the Random Forest we selected 10 features and 350 trees and for the Bayesian model we kept the default settings proposed by the program.

The training continued by creating the final model with 5-fold cross-validation, to keep at least 10 samples per fold. At the end of the training, the performances of the models obtained were compared and the best one was selected.

##### 3.3.3.2. Methods to evaluate and compare alternative solutions

Given the imbalance of data toward improvement, the metric considered the most useful for model evaluation is sensitivity, to maximize the number of true positives (i.e., not improved). In addition, false negatives have a higher cost than false positives: it is less costly to intervene on someone who would not need it, rather than to not intervene on someone who risk a worsening and a potential failure of the surgery.

In addition to the sensitivity, which can provide a misinterpretation of the performance, we also computed the area under the ROC curve.

##### 3.3.3.3. The proposed solution

The first five attributes considered the most important by the feature selection are:

1. The presence of degenerative diseases as an additional pathology;
2. Conservative treatment for more than 12 months;
3. The resolution of peripheral pain as purpose of the intervention;
4. The use of back rigid stabilization techniques;
5. The pre-operative score of the ODI.

**Figure 9:**
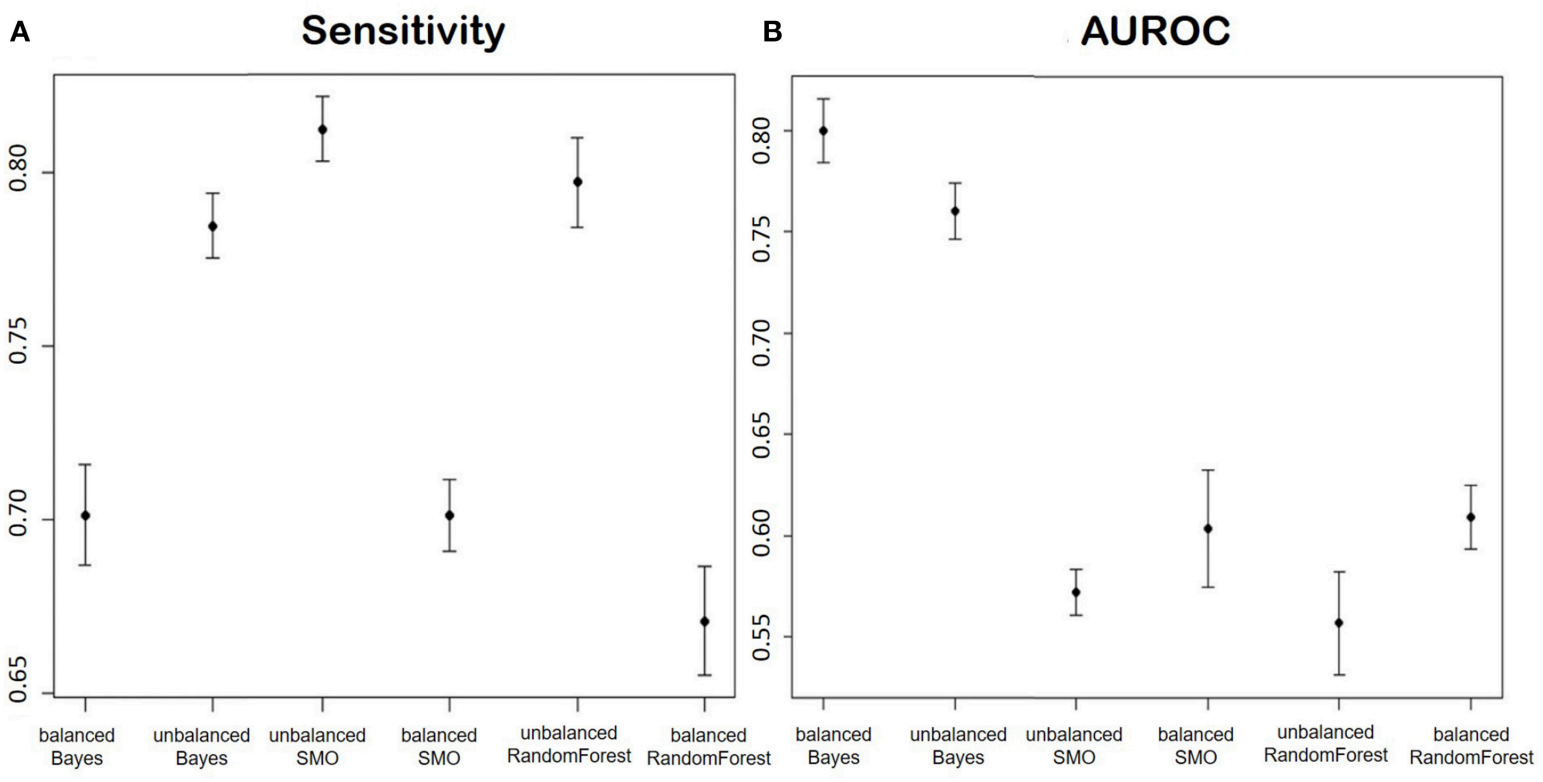
**(A)** Shows the Sensitivity and **(B)** shows AUROC of the models for the prediction of the improvement in terms of ODI in patients of disc herniation. We report in order: Balanced Bayesian model, Unbalanced Bayesian model, Unbalanced Support Vector Machine, balanced Support Vector Machine, unbalanced Random Forest, balanced Random Forest.

Figure 9 shows the averages and confidence intervals produced by the cross-validation of the Sensitivity performance (Figure 9A) and the area under the ROC curve (Figure 9B).

##### 3.3.3.4. Critical evaluation and future works

Considering the two proposed parameters at the same time, the model that had the best performances was the Naïve Bayes trained on an unbalanced dataset. The reason could be that the undersampling applied to balance the classes caused a huge reduction of the number of training data.

The better performance of the Bayesian model could be explained by the nature of the variables: except for the pre-operative ODI, the variables after feature selection were mostly categorical. Bayesian models are often used in texts classification, especially because they are very effective in predicting categorical data.

The main concern in evaluating our solution, in comparison with the others proposed in literature, is that each research group focuses on its own dataset and this makes it difficult to effectively judge if an approach is better than others.

### 3.4. Automatic Patient Cohort Selection

#### 3.4.1. Related Works

**Figure 10:**
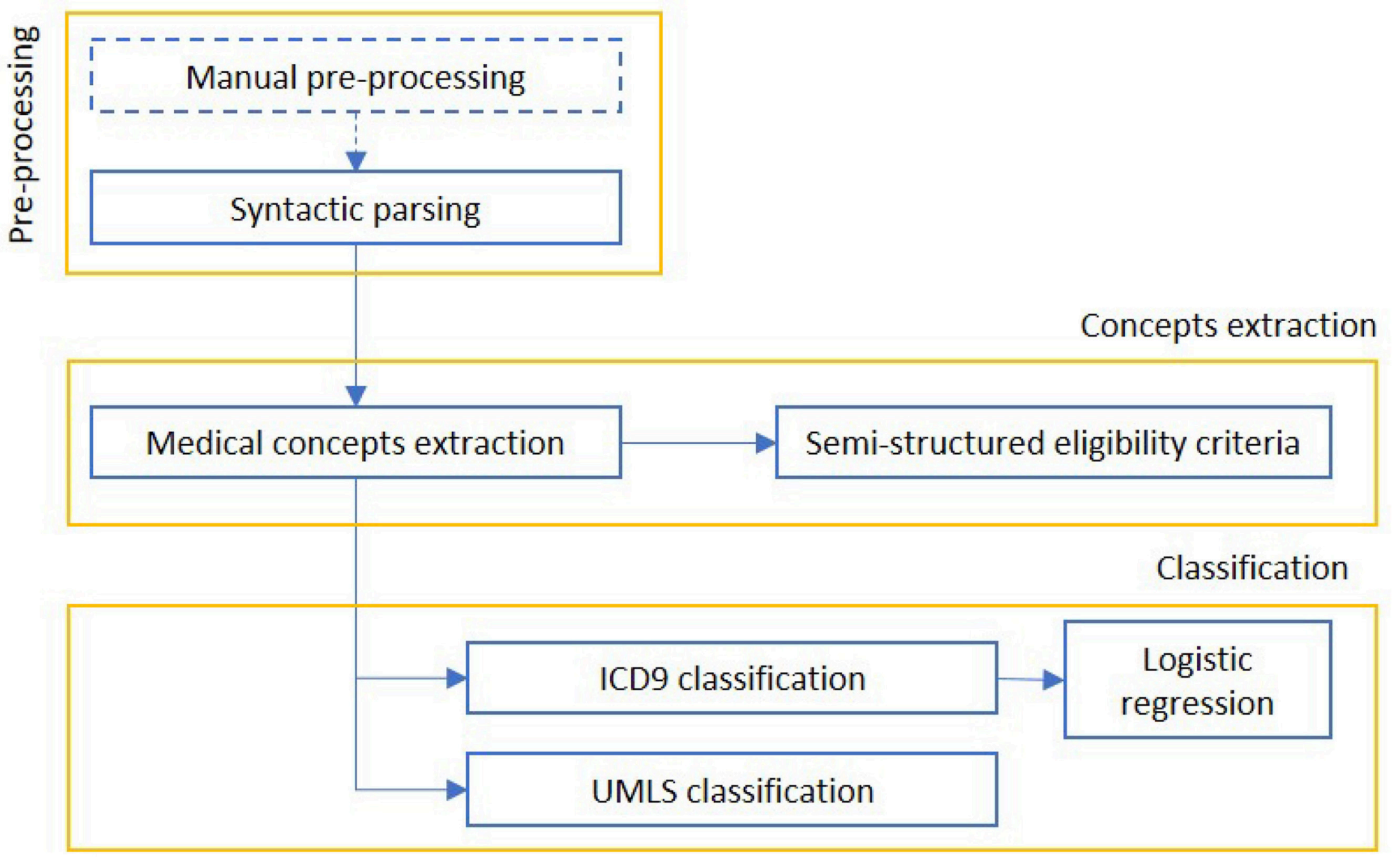
The figure represents a schematization of cohort selection. After an information retrieval process, concepts are mapped to standard medical classifications and used to select the relevant EHR.

We report in Figure 10 a diagram that presents the most relevant methods related to the topic of “cohort selection” as a summary of the methods present in the literature and which we have reported in more detail below.

As above said we will briefly talk about ‘automatic patient cohort selection’ for clinical trials. An important step in clinical trials is the selection of patients that will participate to the tests. As a matter of fact, patients are selected randomly and this is the first problem to obtain valuable results in clinical trials (91). Moreover, another problem is that building cohorts for epidemiologic studies usually relies on a time-consuming and laborious manual selection of appropriate cases.

There are many works that face this topic trying to extract information from EHR to select a specific patients cohort automatically (4, 92–96). The study conducted by Liao et al. (4) showed that the addition of NLP techniques to structured data improved the classification sensitivity compared to algorithms that use only structured data.

Another example (despite this is not strictly related to clinical trials) is the research by Sada et al. (97) that tried to identify patients with HCC (hepatocellular cancer) using directly EHRs. Reports were first manually classified as diagnostic of HCC or not, then NLP techniques by the Automated Retrieval Console (ARC) were implemented to perform a classification of the documents using the Clinical Text Analysis and Knowledge Extraction System. The results showed that the classification performance improved using a combined approach of ICD9 codes and NLP techniques.

EMRs can be used to enable large-scale clinical studies. The aim of the research conducted by Kumar et al. (98) was to create an EMR cohort of T2D (type 2 diabetes) patients. NLP was performed on narrative notes using the previously described platform called cTAKES that extracts medical concepts. Then, a logistic regression algorithm was implemented to perform a classification using codified data (ICD9) and narrative NLP data. The results showed a good identification of patients' cohort, with a 97% of specificity and 0.97 of positive predictive value (PPV).

Clinical research eligibility criteria specify the medical, demographic, or social characteristics of eligible clinical research volunteers. Their free-text format remains a significant barrier to computer-based decision support for electronic patient eligibility determination. EliXR is a semi-automated approach, developed by Weng et al. (99) that standardizes eligibility concept encoding, through UMLS coding, and allows syntactic parsing to reduce complexity of patterns. The generated labels were used to generate semi-structured eligibility criteria.

## 4. Discussion

What's remarkable is that we can develop complex models depending on the quality and the quantity of the available data. Starting from unstructured data (e.g., discharge letters) we have seen how, using a basic model, it is possible to structure it aiming to extract useful information (e.g., for sentiment analysis). Then, we found how to extract concepts, even tailored, with a moderate effort in word tagging (e.g., transfer learning in section 3.2.3). Finally, in the case we already had structured data, we have seen how it is possible to create predictive models on the outcomes. The information extraction process may be time-consuming, but make unstructured clinical data usable seems to give promising results.

An important consideration is the need of reliable data. Data variability, that makes the secondary use of collected data a burdensome work, could be avoided. For example spell checking features should be introduced on medical software to avoid typing errors.

The considerable variability found among the more formal texts is damaging knowledge extraction. In other cases, however, we must remember that variability is a wealth. Think of the example of patients' comments, where different lexical nuances could lead to different sentiment, useful to understand the causes of an illness.

Concerning the methods we used, we stressed once again the importance of “No Free Lunch Theorem” (100). It stated that we can find the best solutions of very similar problems with different techniques or approaches. For example, balancing the training set sometimes improves the classification performance (e.g., in the classification of the discharge letters), while in others it worsens it (e.g., in the outcomes prediction). We also highlight the importance of data preprocessing that is a large part of creation-modeling pipeline, for example the noise reduction, the creation of bag-of-words, the feature selection and the removal of outliers.

## 5. Future Works and Conclusions

As said in the Introduction, Big Data does not only refer to the quantity of data but also on quality (veracity) and variety. Our contribution has mainly focused on these two aspects. We have surveyed the most recent and relevant contributions that focus on how to cope with, and indeed leverage, content diversity, namely the coexistence of structured and unstructured data in the same record pertaining the same patient and care trajectory, in order to improve the quality of EHR data, and the related processes.

Unstructured data is the content that caregivers and patients produce in each phase of the care trajectory and report as free-text in a number of documents, like anamnestic notes, medical and nurse diaries, surgical records, discharge letters, discharge reports, as well as side comments, notes extending patient-reported outcome measures, and the like. Current research focusing on these information resources has so far focused on mainly two tasks: (i) to extract entities and values from this varied content, and match these data with the structured data natively available in the same record, in order to detect possible discrepancies, anomalies, and inconsistencies (even at semantic level) between these two complementary sources of patient information, as well as to find opportunities for faster and less error-prone data entry (e.g., context-driven check lists, *ad-hoc* templates, auto complete features); (ii) evaluate the “sentiment” of this content, as a proxy of the context that is not reported in codified and structured manner, and assess its value in predictive and prognostic (machine-learning-based) models which are aimed at predicting complication risk and mental and physical scores at specific steps in the post-operative follow-up trajectory. In regard to both these tasks, this survey discusses the main contributions that can inform future works and achievements. However, we have also reported about the application of quick-and-dirty NLP techniques to the “not so small” data of the electronic medical registry of spine surgery and joint replacement procedures that since 2016 has been adopted at the IRCCS Orthopedic Institute Galeazzi, one of the main Italian teaching hospitals specialized in musculoskeletal disorders. It is especially this second contribution of this work that emphasizes how it is still difficult to address the questions raised by this special issue: and in particular whether NLP can already be considered disruptive in medicine, or it is still in its infancy, despite the great potential discussed in this review.

As regarding the empirical works, we provided the reader with some simple and affordable pipelines, which demonstrate the feasibility of reaching literature performance levels with a single institution non-English dataset. In such a way, we bridged literature and real world needs, performing a step further toward the revival of notes fields.

We observed a wide variability on the number of available papers across the range of topics we covered in our literature review. The most popular topic is “information extraction” so far, while “sentiment analysis” in the ambit of predictive analysis is the least popular, indicating a need for further research in this direction. We cannot predict which topic will attract more interest in the medical field, not even in the short term (let alone, mid or long term). For instance, in regard to the former topic above, a recent deep learning technique (generative adversarial networks) has been successfully applied to create de-identified and standardized (with respect to abbreviations and local shorthands) text and capture data in free text notes (28). This is a new approach that looks promising and that will probably be adopted in an increasing number of settings. We can expect that all of the above topics will attract some new research, and this review has been mainly aimed at providing convenient access to the main trends that have emerged in digital medicine so far in the high-potential field of the processing of unstructured text in medical records.

## Author Contributions

All authors contributed to the manuscript, in various ways. FC conceived the project, the main conceptual ideas and proof outline. FC also wrote both the introduction and part of the conclusions and supervised the project providing critical feedback. MA and AC collected the sources for the literature review, and analyzed them according to the proof outline. LD and AS collected the IOG data, cleansed these data, selected the NLP methods, performed the analytic calculations, reported the results and drafted a preliminary discussion of the empirical part of the study. MA wrote most of the literature review and integrated the review and empirical part together into the final manuscript. AC verified the analytical methods and revised the manuscript's content thoroughly. All authors contributed to the interpretation of the results.

### Conflict of Interest Statement

MA was employed by company K-Tree Srl. LD was employed by company Link-Up Datareg. AC was employed by company K-Tree Srl. AS was employed by company Link-Up Datareg. The remaining authors declare that the research was conducted in the absence of any commercial or financial relationships that could be construed as a potential conflict of interest.

## Footnotes

^1^https://www.zdnet.com/article/googles-deep-learning-system-aims-to-tame-electronic-health-records/

^2^https://www.zdnet.com/article/aws-launches-comprehend-medical-applies-natural-language-processing-to-medical-records/

^3^https://towardsdatascience.com/https-medium-com-vishalmorde-humanizing-customer-complaints-using-nlp-algorithms-64a820cef373 (accessed February 13, 2019).

^4^http://hdl.handle.net/11299/137703 (accessed August 01, 2017).

^5^https://www.definedcrowd.com/

^6^https://github.com/pazqo/scalaWords (accessed September 18, 2018).

^7^http://creativyst.com/Doc/Articles/SoundEx1/SoundEx1.htm (accessed March 01, 2019).

^8^http://aspell.net/metaphone/ (accessed March 01, 2019).

^9^https://www.rdocumentation.org/packages/glmnet/versions/2.0-16/topics/glmnet (accessed October 10, 2018).

^10^https://spacy.io/ (accessed October 10, 2018).

^11^https://explosion.ai/blog/deep-learning-formula-nlp (accessed October 10, 2018).

^12^https://spacy.io/api/annotation. (accessed October 10, 2018).

^13^https://github.com/cjhutto/vaderSentiment (accessed October 10, 2018).

^14^http://cs.stanford.edu/people/alecmgo/trainingandtestdata.zip (accessed October 10, 2018).
